# National

**Published:** 1897-05

**Authors:** 


*National.* Secretary Wilson, of the Department of Agriculture, has determined upon plans looking to the eradication of hog-cholera. Several centres of work in this direction will be established, where the coöperation of the people may be secured. The Government will assume the entire charge and the expense incidental thereto, and pay for all hogs killed at their pork-value. Careful microscopic and chemical examinations and analyses will be followed with the hope that results may be obtained which will warrant a general effort in this direction another year.

